# Two Functional Variants of *IRF5* Influence the Development of Macular Edema in Patients with Non-Anterior Uveitis

**DOI:** 10.1371/journal.pone.0076777

**Published:** 2013-10-07

**Authors:** Ana Márquez, María Carmen Cénit, Miguel Cordero-Coma, Norberto Ortego-Centeno, Alfredo Adán, Alejandro Fonollosa, David Díaz Valle, Esperanza Pato, Ricardo Blanco, Joaquín Cañal, Manuel Díaz-Llopis, Enrique de Ramón, María José del Rio, José Luis García Serrano, Joseba Artaraz, José Manuel Martín-Villa, Víctor Llorenç, Marina Begoña Gorroño-Echebarría, Javier Martín

**Affiliations:** 1 Instituto de Parasitología y Biomedicina López-Neyra, IPBLN, CSIC, Granada, Spain; 2 Ophthalmology Department, Hospital de León, León, Spain; 3 Internal Medicine Department, Hospital Clínico San Cecilio, Granada, Spain; 4 Ophthalmology Department, Hospital Clínic, Barcelona, Spain; 5 Ophthalmology Department, Hospital de Cruces, Bilbao, Spain; 6 Ophthalmology Department, Hospital Clínico San Carlos, Madrid, Spain; 7 Rheumatology Department, Hospital Clínico San Carlos, Madrid, Spain; 8 Rheumatology Department, Hospital Marqués de Valdecilla, IFIMAV, Santander, Spain; 9 Ophthalmology Department, Hospital Marqués de Valdecilla, IFIMAV, Santander, Spain; 10 Ophthalmology Department, Hospital La Fe, Valencia, Spain; 11 Internal Medicine Department, Hospital Carlos Haya, Málaga, Spain; 12 Ophthalmology Department, Hospital Carlos Haya, Málaga, Spain; 13 Ophthalmology Department, Hospital Clínico San Cecilio, Granada, Spain; 14 Immunology Department, Facultad de Medicina, Universidad Complutense de Madrid, Madrid, Spain; 15 Ophthalmology Department, Hospital Principe de Asturias, Alcalá de Henares, Spain; Oregon Health & Science University, United States of America

## Abstract

**Objective:**

Interferon (IFN) signaling plays a crucial role in autoimmunity. Genetic variation in interferon regulatory factor 5 (IRF5), a major regulator of the type I interferon induction, has been associated with risk of developing several autoimmune diseases. In the current study we aimed to evaluate whether three sets of correlated *IRF5* genetic variants, independently associated with SLE and with different functional roles, are involved in uveitis susceptibility and its clinical subphenotypes.

**Methods:**

Three *IRF5* polymorphisms, rs2004640, rs2070197 and rs10954213, representative of each group, were genotyped using TaqMan® allelic discrimination assays in a total of 263 non-anterior uveitis patients and 724 healthy controls of Spanish origin.

**Results:**

A clear association between two of the three analyzed genetic variants, rs2004640 and rs10954213, and the absence of macular edema was observed in the case/control analysis (*P*_*FDR*_=5.07E-03, OR=1.48, CI 95%=1.14-1.92 and *P*_*FDR*_=3.37E-03, OR=1.54, CI 95%=1.19-2.01, respectively). Consistently, the subphenotype analysis accordingly with the presence/absence of this clinical condition also reached statistical significance (rs2004640: *P*=0.037, OR=0.69, CI 95%=0.48-0.98; rs10954213: *P*=0.030, OR=0.67, CI 95%=0.47-0.96), thus suggesting that both *IRF5* genetic variants are specifically associated with the lack of macular edema in uveitis patients.

**Conclusion:**

Our results clearly showed for the first time that two functional genetic variants of *IRF5* may play a role in the development of macular edema in non-anterior uveitis patients. Identifying genetic markers for macular edema could lead to the possibility of developing novel treatments or preventive therapies.

## Introduction

Uveitis is a general term used to describe a diverse group of clinical entities with a variety of etiologies. It is one of the leading causes of blindness worldwide accounting for about 10% of the severe visual handicap [1,2]. Uveitis patients are frequently classified according to the anatomical location of the inflammation into anterior uveitis (AU), the most common form, intermediate uveitis (IU), posterior uveitis (PU) and panuveitis or also called diffuse uveitis (PAN) [3].

Non-infectious uveitis is believed to have an autoimmune component [4,5]. Studies in human and animal models have suggested that autoimmune uveitis presents a complex inheritance pattern with multiple genetic and environmental factors involved in the appearance and progression of this condition [6,7].

The genetic predisposition to develop uveitis has long been investigated. The association between certain HLA alleles, particularly HLA-A29 in birdshot chorioretinopathy and HLA-B27 in anterior uveitis, is well known [8,9]. However, HLA accounts for only a small part of the genetic predisposition to immune-mediated uveitis.

Outside the HLA region, several genes have been proposed to play a role in the development of diseases involving uveitis; however only a few *loci* have been confirmed as genetic risk factors, such as *IL23R/IL12RB2* or *IL10* in Behçet’s disease (BD) [10,11], and, currently, the overall underlying mechanisms of the uveitis induction remain unclear.

The type I interferons (IFNs) are signalling molecules involved in both innate and adaptive immunity. The IFN pathway has been postulated to play a crucial role in autoimmune diseases. Specifically, several single nucleotide polymorphisms (SNPs) on the interferon regulatory factor 5 (IRF5) gene, a major regulator of the type I IFN induction and critical for the production of pro-inflammatory cytokines, have been associated with different autoimmune conditions such as rheumatoid arthritis, systemic lupus erythematosus (SLE), multiple sclerosis, systemic sclerosis, inflammatory bowel disease and Sjöegren’s syndrome [12–17]. In SLE, three independent sets of correlated *IRF5* variants that provide statistically independent evidence for association have been reported [18]. Polymorphisms in each of these groups seem to have different functional consequences such as alteration of a consensus splice donor site allowing expression of an alternative exon 1, creation of an early polyadenylation site that leads to a shorter isoform and alteration of the protein stability.

It has been suggested that autoimmune uveitis shares different genetic factors with other autoimmune disorders [19]. The aim of our study was to analyze three SNPs, representative of each group of variants independently associated with SLE, in order to explore the role of *IRF5* in uveitis susceptibility and its clinical subgroups.

## Patients and Methods

### Ethics statement

A subject’s written consent was obtained according to the declaration of Helsinki, and the design of the work was approved by the Ethics Committee of Granada (Spain). The Ethics Committees of the Hospital de León (León), Hospital Universitario Príncipe de Asturias (Alcalá de Henares), Hospital de Cruces (Bilbao), Hospital Clinic (Barcelona), Hospital Clínico San Carlos (Madrid), Hospital Marqués de Valdecilla (Santander), Hospital Universitario La Fe (Valencia), Hospital Clínico San Cecilio (Granada) and Hospital Carlos Haya (Málaga) also approved the study.

### Patients

A total of 263 patients with non-infectious uveitis, excluding the exclusively anterior uveitis forms as well as uveitis associated with systemic immune-mediated diseases except Vogt-Koyanagi-Harada syndrome, and 724 ethnically matched healthy controls, all of them from Spain, were included in the present study.

In addition, to examine whether the selected SNPs might influence the different clinical manifestations of the disease, uveitis patients were subdivided according to different characteristics included in Table **1**. The intraocular inflammation seen in patients included intermediate uveitis (24.0%), posterior uveitis (51.7%), and panuveitis (24.3%). Macular edema was diagnosed by biomicroscopy and optical coherence tomography (Cirrus HD-OCT, Carl Zeiss Meditec, Inc). Scans were obtained using the 512 x 128 scan pattern. Fluorescein angiography (FA) was performed to rule out macular edema in doubtful cases. Macular edema was defined as a macular thickness greater than 250 µm with the presence of intraretinal cysts demonstrated by OCT [20], and/or the presence of retinal angiographic leakage in the central macula area for those patients in which FA was performed.

**Table 1:** Clinical and demographic features of uveitis patients.

**General characteristics of uveitis patients**	**Uveitis (n=263)**	**Controls (n=724)**
Female (%)	146 (55.5)	481 (66.4)
Age (mean ± SD)	48.3 ± 16.4	49.1 ± 12.5
Intermediate Uveitis (%)	63 (24.0)	-
Posterior Uveitis (%)	136 (51.7)	-
Panuveitis (%)	64 (24.3)	-
Bilaterality (%)	206 (78.3)	-
Vitritis (%)	177 (67.3)	-
Macular edema (%)	121 (46.0)	-
Retinal vasculitis (%)	116 (44.1)	-
Choroidal neovascularization (%)	25 (9.51)	-

### Genotyping

Genomic DNA was extracted from peripheral white blood cells and saliva, using standard procedures. Three SNPs, rs2004640, rs2070197 and rs10954213, located on the *IRF5* gene, were genotyped using a pre-designed TaqMan® allelic discrimination assay in a 7900HT Real-Time polymerase chain reaction (PCR) System from Applied Biosystems (Foster City, CA, USA). The genotype call rate was 99% for the tested genetic variants. In addition, randomly selected samples were genotyped twice to verify the genotyping accuracy. The 99% of the genotypes were identical.

### Statistical analysis

We carried out a case-control study using Plink (v1.07) (http://pngu.mgh.harvard.edu/purcell/plink/). The genotype, allele and carrier frequencies were compared between patients, subgroup of patients and controls applying χ^2^ test and/or Fisher’s exact test when necessary (when the expected frequencies were lower than 5). Odds ratios (OR) and 95% confidence intervals (CI) were obtained according to Woolf’s method. Since different polymorphisms were analyzed in the current study, *the* Benjamini & Hochberg (1995) step-up false discovery rate (FDR) control correction for multiple testing was applied to the *P*-values. *P*-values after FDR correction lower than 0.05 were considered as statistically significant.

Dependency of association between the studied genetic variants was determined by conditional logistic regression analysis implemented in PLINK, and the allelic combinations were tested using PLINK and Haploview (V. 4.2).

To analyze whether allelic combinations would better explain the possible association than the genetic variants independently, we compared the goodness of fit of both models using PLINK. For that purpose, we calculated the deviance (defined as -2 x the log likelihood), which follows a χ^2^ distribution, to assess the significance of the improvement in fit. If statistically significant differences in the improvement of fit were observed when the haplotype effect was considered, we assumed that this model was more informative explaining the putative association.

## Results

No statistically significant deviation from Hardy-Weinberg equilibrium (P> 0.01) was observed for every SNP in the controls set and the frequencies of the analyzed SNPs were in agreement with the data of the HapMap project.

### Allele test

As shown in Table **2**, when genotype and allelic frequencies were compared between uveitis patients and controls, a trend of association of rs2004640 and rs10954213 with uveitis was found. However, the stratification of uveitis patients accordingly with the presence/absence of the different clinical characteristics, showed in Table **1**, evidenced that these signals were due to a specific association with patients without macular edema (rs2004640: *P*_*FDR*_=5.07E-03, OR=1.48, CI 95%=1.14-1.92; rs10954213: *P*_*FDR*_=3.37E-03, OR=1.54, CI 95%=1.19-2.01). Consistently, statistically significant differences were observed when comparing uveitis patients with and without this trait (rs2004640: *P*=0.037, OR=0.69, CI 95%=0.48-0.98; rs10954213: *P*=0.030, OR=0.67, CI 95%=0.47-0.96) (Table **2**).

**Table 2:** Genotype and minor allele frequencies of *IRF5* genetic variants in uveitis patients and healthy controls.

			**Genotype, N (%)**				**Allele test**		
**SNP**	**1/2**	**Subgroup (N)**	**1/1**	**1/2**	**2/2**	**MAF (%)**	*P*-value*	*P*_FDR_**	OR [CI 95%]***
rs2004640 a	G/T	Controls (n=723)	135 (18.67)	356 (49.24)	232 (32.09)	43.29			
		Uveitis (n=258)	58 (22.48)	132 (51.16)	68 (26.36)	48.06	0.0613	0.0919	1.21 [0.99-1.48]
		With edema (n=119)	25 (21.01)	54 (45.38)	40 (33.61)	43.70	0.9068	0.9068	1.02 [0.77-1.34]
		Without edema (n=134)	32 (23.88)	78 (58.21)	24 (17.91)	52.99	**3.38E-03**	**5.07E-03**	1.48 [1.14-1.92]
rs2070197 b	C/T	Controls (n=723)	9 (1.24)	113 (15.63)	601 (83.13)	9.06			
		Uveitis (n=261)	1 (0.38)	35 (13.41)	225 (86.21)	7.09	0.1671	0.1671	0.77 [0.52-1.12]
		With edema (n=120)	0 (0.00)	17 (14.17)	103 (85.83)	7.08	0.3164	0.9068	0.77 [0.45-1.29]
		Without edema (n=136)	1 (0.74)	17 (12.50)	118 (86.76)	6.99	0.2662	0.2662	0.75 [0.46-1.24]
rs10954213 c	G/A	Controls (n=723)	88 (12.17)	323 (44.67)	312 (43.15)	34.51			
		Uveitis (n=261)	48 (18.39)	112 (42.91)	101 (38.70)	39.85	0.0293	0.0880	1.26 [1.02-1.55]
		With edema (n=120)	19 (15.83)	47 (39.17)	54 (45.00)	35.42	0.7843	0.9068	1.04 [0.78-1.39]
		Without edema (n=136)	29 (21.32)	64 (47.06)	43 (31.62)	44.85	**1.12E-03**	**3.37E-03**	1.54 [1.19-2.01]

* All P-values have been calculated for the allelic model. ** Benjamini & Hochberg (1995) step-up FDR control. *** Odds ratio for the minor allele.

^a^ Uveitis with edema vs. uveitis without edema: *P*= 0.037, OR (CI 95%)=0.69 (0.48-0.98); ^b^ Uveitis with edema vs. uveitis without edema: *P*=0.966, OR (CI 95%)=1.02 (0.52-2.00); ^c^ Uveitis with edema vs. uveitis without edema: *P* = 0.030, OR (CI 95%)=0.67 (0.47-0.96);

### Conditional logistic regression

Since an independent effect of the two associated SNPs has been reported, we decided to perform pairwise conditioning analysis to test whether there could be any dependence among them (Table **S1**). However, both SNPs lost the statistical significance after conditioning to each other.

### Haplotype analysis

We also carried out the allelic combination analysis for studied *IRF5* polymorphisms (Figure **S1** shows the linkage disequilibrium between the three analyzed polymorphisms). This analysis also showed a phenotype-specific association of *IRF5* with the absence of macular edema (Table **3**) but not with the global disease (data not shown). Two associated haplotypes, containing the risk and protective alleles of the two associated SNPs, were found in the comparison between patients without macular edema and controls (rs2004640*G-rs2070197*T-rs10954213*G: *P*_*FDR*_=0.011, OR=1.51, CI 95%=1.16-1.98; rs2004640*T-rs2070197*T-rs10954213*A: *P*_*FDR*_=0.020, OR=0.70, CI 95%=0.53-0.92). When patients with and without edema were compared, an association of the rs2004640*G-rs2070197*T-rs10954213*G haplotype was also evident (*P*=6.69E-03, OR=0.59, CI 95%=0.41-0.86).

**Table 3:** Different allelic combinations of the *IRF5* genomic region according to the presence/absence of macular edema in uveitis patients.

				**Without macular edema vs. controls**			**With vs. Without macular edema**		
**Allelic combination**	**Freq. With edema**	**Freq. Without edema**	**Freq. Controls**	**P-value**	**P_FDR_**	**OR [95% CI]**	**P-value**	P_FDR_*	**OR [95% CI]**
GTG	0.279	0.397	0.304	**2.22E-03**	**0.011**	1.51 [1.16-1.98]	**6.69E-03**	**0.033**	0.59 [0.41-0.86]
TTA	0.413	0.349	0.435	**8.06E-03**	**0.020**	0.70 [0.53-0.92]	0.108	0.271	1.31 [0.91-1.87]
GTA	0.163	0.132	0.129	0.887	0.351	1.05 [0.71-1.53]	0.451	0.375	1.27 [0.78-2.07]
TCA	0.071	0.070	0.091	0.281	0.351	1.00 [0.83-1.20]	0.939	0.375	1.00 [0.78-1.28]
TTG	0.075	0.051	0.041	0.491	0.491	1.32 [0.73-2.37]	0.375	0.375	1.47 [0.72-2.99]

Order of the SNPs: rs2004640*G/T │rs2070197*C/T │rs10954213*A/G

* Benjamini & Hochberg (1995) step-up FDR control

OR, odds ratio

When comparing the haplotype model with the independent SNP model, we did not observe a statistically significant improvement of the goodness of fit compared to rs2004640 (without macular edema vs. controls: likelihood *P-value*=0.073; with vs. without macular edema: likelihood *P-value*=0.071) or rs10954213 (without macular edema vs. controls: likelihood *P-value*=0.180; with vs. without macular edema: likelihood *P-value*=0.061) individually.

## Discussion

IRF5 is member of a family of transcription factors that play a crucial role in the control of inflammatory and immune responses. Several studies have established that IRF5 is involved in the production of type I IFNs and it is critical for the production of the pro-inflammatory cytokines TNF-α, IL-12 and IL-6, following Toll-like receptor signaling [21]. Recently the role of the type I interferon pathway in the development of autoimmunity has become increasingly important. Additionally, several genetic studies have identified *IRF5* genetics variants associated with an increased risk of several autoimmune diseases [12–17].

Three groups of correlated *IRF5* SNPs, designated as Groups 1, 2 and 3, independently associated with SLE and with different functional roles have been described [18]. Group 1 includes SNPs tagging a 30-bp in-frame INDEL variant of exon 6 that alters protein stability; the association of the Group 2 SNPs seems to be explained by the T allele of rs2004640 which allows the expression of an alternative isoform 1B and is associated with significantly higher levels of *IRF5* expression; and finally, the association of the Group 3 SNPs is probably due to the rs10954213 A allele that creates an early polyadenylation site leading to a higher *IRF5* expression [18,22,23]. All the risk alleles of these polymorphisms segregate in a strongly associated haplotype tagged by the C allele of the rs2070197, belonging to Group 1.

In the present study we analyzed one tagging SNP of each group of independent signals, rs2004640, rs2070197 and rs10954213. No association of any of these polymorphisms with the global disease was evident, however, our results clearly showed that two of the three analyzed variants, rs2004640 and rs10954213, influence the development of macular edema in uveitis patients, supported by a high statistical power; as shown in Table **S2**, the statistical power of our study is higher than 80% to detect the observed odds ratio (OR=1.5). It was not possible to determine whether these two genetic variants represent independent signals in uveitis, as previously reported in other autoimmune diseases. The fact that the conditional logistic regression analysis showed no conclusive results could be due to a reduced statistical power. On the other hand, no additive effect among these two functional *IRF5* polymorphisms was evident, since the model considering a haplotype effect did not better explain the observed association than that of the independent SNPs. Nevertheless, this should be taken with caution, because a lack of statistical power could also represent a limitation in this analysis; indeed, the comparison of the goodness of fit of both models almost reached statistical significance. Regarding rs2070197, we found no evidence of association with non-anterior uveitis or clinical subphenotypes. However, the statistical power of this analysis was compromised due to the low frequency of the minor allele and, therefore, weak effects were hardly detected.

Opposed to our results, a previous study performed in Han Chinese population [24] failed to show association between two *IRF5* polymorphisms, rs2280714 and rs752637, and BD. It is important to note that both belong to Group 3 and are highly correlated with rs10954213 (r^2^>0.8); therefore, these genetic variants have been covered in our study.

In uveitis, macular edema forms a major cause of visual impairment and blindness [2]. The exact pathogenesis of this trait in patients with uveitis is not clear but the cause of pathologic fluid retention and protein accumulation within the retina seems to be a disruption of the blood retinal barrier (BRB) formed by the zonulae occludentes and adhaerentes of the retinal pigment epithelial cells (outer barrier) and endothelial cells of retinal vessels (inner barrier). Intraocular inflammation may lead to the disturbance of the BRB via the release of local inflammatory mediators. It has been shown that IL-6 may indirectly cause an increase of vascular permeability and angiogenesis by inducing the expression of VEGF (vascular endothelial growth factor), a major known factor in angiogenesis and vasopermeability in the eye [25]. Elevated levels of intraocular IL-6 and TNF-α, whose expression is upregulated by IRF5, have been reported in patients with intermediate uveitis, especially in those with macular edema [26].

Our data are consistent with these findings. Taking into account that the frequency of the rs2004640*T and rs10954213*A alleles is lower in the group of patients without macular edema than in those presenting this trait, it could be speculated that patients without edema present decreased IRF5 levels and, therefore, lower levels of inflammatory cytokines involved in the development of macular edema, including TNF- α and IL-6.

Interestingly, several studies have reported that the treatment with type I IFNs (IFN-β-1 and IFN-α-2a) leads to complete or partial resorption of macular edema in a high percentage of uveitis patients [27,28], thus supporting the key role of the IFN signaling in this trait.

In conclusion, we show for a first time that two genetic variants within the *IRF5* region influence the macular edema development in patients with non-anterior uveitis. Functional studies will be necessary to clarify the implication of the *IRF5* alleles in the pathophysiology of this trait.

## Supporting Information

Figure S1 **Linkage disequilibrium between the analyzed *IRF5* genetic variants.**
(DOCX)

Table S1 **Conditional logistic regression analysis for rs2004640 and rs10954213 IRF5 genetic variants.**
(DOCX)

Table S2 **Statistical power of the comparison between uveitis patients without macular edema and controls for each analyzed *IRF5* genetic variants at the 5% significance level.**
(DOCX)
