# Supplementary material for: Two Functional Variants of IRF5 Influence the Development of Macular Edema in Patients with Non-Anterior Uveitis
Source: PLoS One. 2013 Oct 7;8(10):e76777. doi: 10.1371/journal.pone.0076777 (PMC3792064; doi:10.1371/journal.pone.0076777)
Supplement: Figure S1 — Linkage disequilibrium between the analyzed IRF5 genetic variants. (DOCX) [file pone.0076777.s001.docx]

**Figure S1**. Linkage disequilibrium between the analyzed *IRF5* genetic variants.


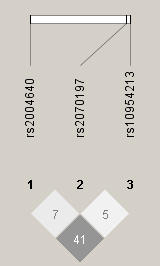


r^2^ values are showed
