# Supplementary material for: Two Functional Variants of IRF5 Influence the Development of Macular Edema in Patients with Non-Anterior Uveitis
Source: PLoS One. 2013 Oct 7;8(10):e76777. doi: 10.1371/journal.pone.0076777 (PMC3792064; doi:10.1371/journal.pone.0076777)
Supplement: Table S1 — Conditional logistic regression analysis for rs2004640 and rs10954213 IRF5 genetic variants. (DOCX) [file pone.0076777.s002.docx]

**Table S1.** Conditional logistic regression analysis for rs2004640 and rs10954213 *IRF5* genetic variants.

|  | **Without edema vs. controls** | | | **With edema vs. without edema** | | |
| --- | --- | --- | --- | --- | --- | --- |
|  | P-value | P-value  add to rs10954213 | P-value  add to rs2004640 | P-value | P-value  add to rs10954213 | P-value  add to rs2004640 |
| rs2004640 | **3.38E-03** | 0.225 | N/A | **0.037** | 0.289 | N/A |
| rs10954213 | **1.12E-03** | N/A | 0.109 | **0.030** | N/A | 0.252 |
