# Supplementary material for: Two Functional Variants of IRF5 Influence the Development of Macular Edema in Patients with Non-Anterior Uveitis
Source: PLoS One. 2013 Oct 7;8(10):e76777. doi: 10.1371/journal.pone.0076777 (PMC3792064; doi:10.1371/journal.pone.0076777)
Supplement: Table S2 — Statistical power of the comparison between uveitis patients without macular edema and controls for each analyzed IRF5 genetic variants at the 5% significance level. (DOCX) [file pone.0076777.s003.docx]

**Table S2.** Statistical power of the comparison between uveitis patients without macular edema and controls for each analyzed *IRF5* genetic variants at the 5% significance level.

| **SNP** | **OR=1.1** | **OR=1.2** | **OR=1.3** | **OR=1.4** | **OR=1.5** |
| --- | --- | --- | --- | --- | --- |
| **rs2004640** | 0.11 | 0.28 | 0.51 | 0.72 | 0.86 |
| **rs2070197** | 0.06 | 0.11 | 0.20 | 0.31 | 0.44 |
| **rs10954213** | 0.10 | 0.26 | 0.48 | 0.69 | 0.84 |

SNP, single-nucleotide polymorphism; OR, odds ratio.
